# Supplementary figures and images for: The association between maternal prenatal folic acid and multivitamin supplementation and autism spectrum disorders in offspring: An umbrella review
Source: PLoS One. 2025 Nov 18;20(11):e0334852. doi: 10.1371/journal.pone.0334852 (PMC12626298; doi:10.1371/journal.pone.0334852)

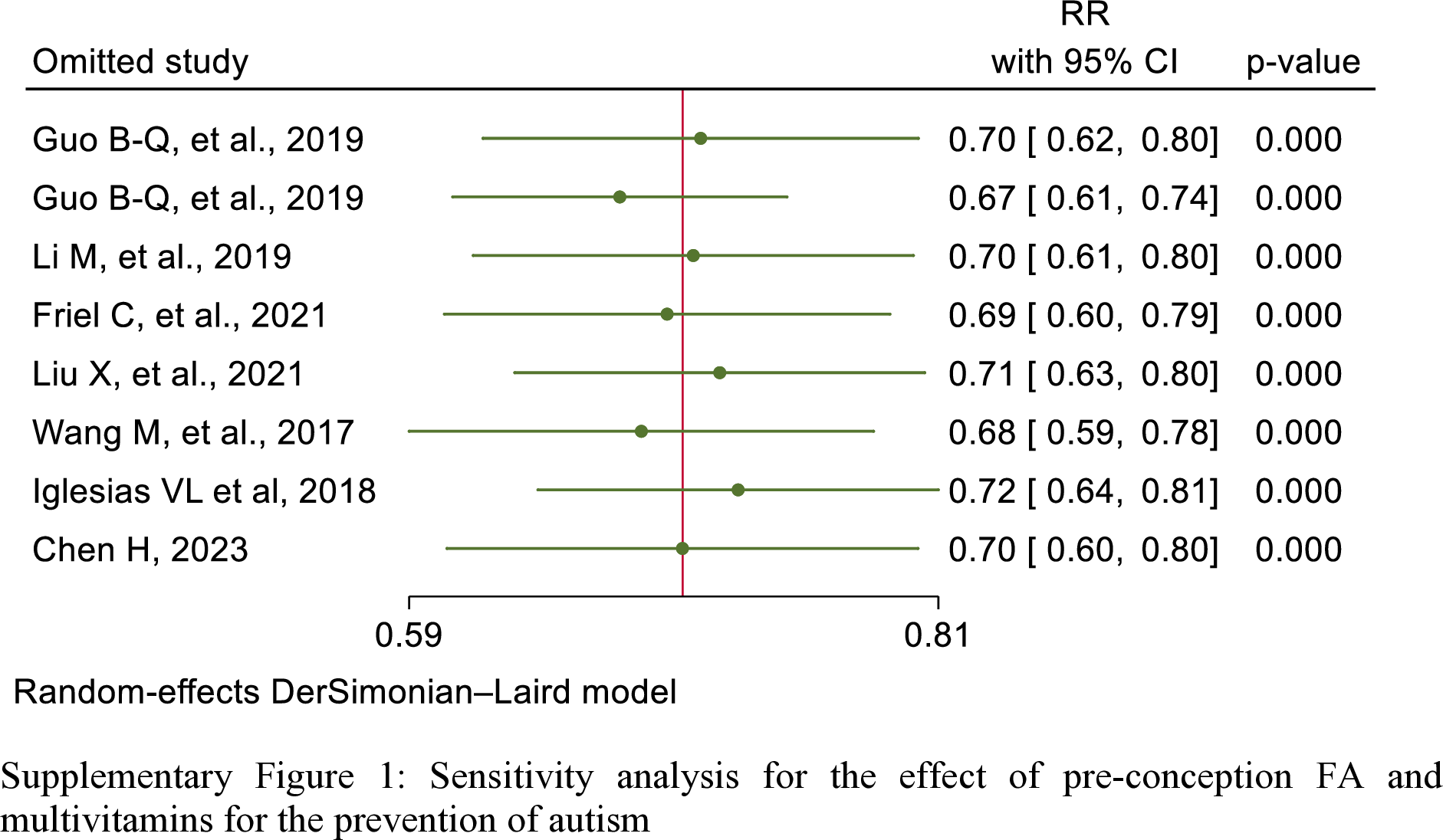

Supplement: S1 Fig — (TIF) [file pone.0334852.s001.tif]

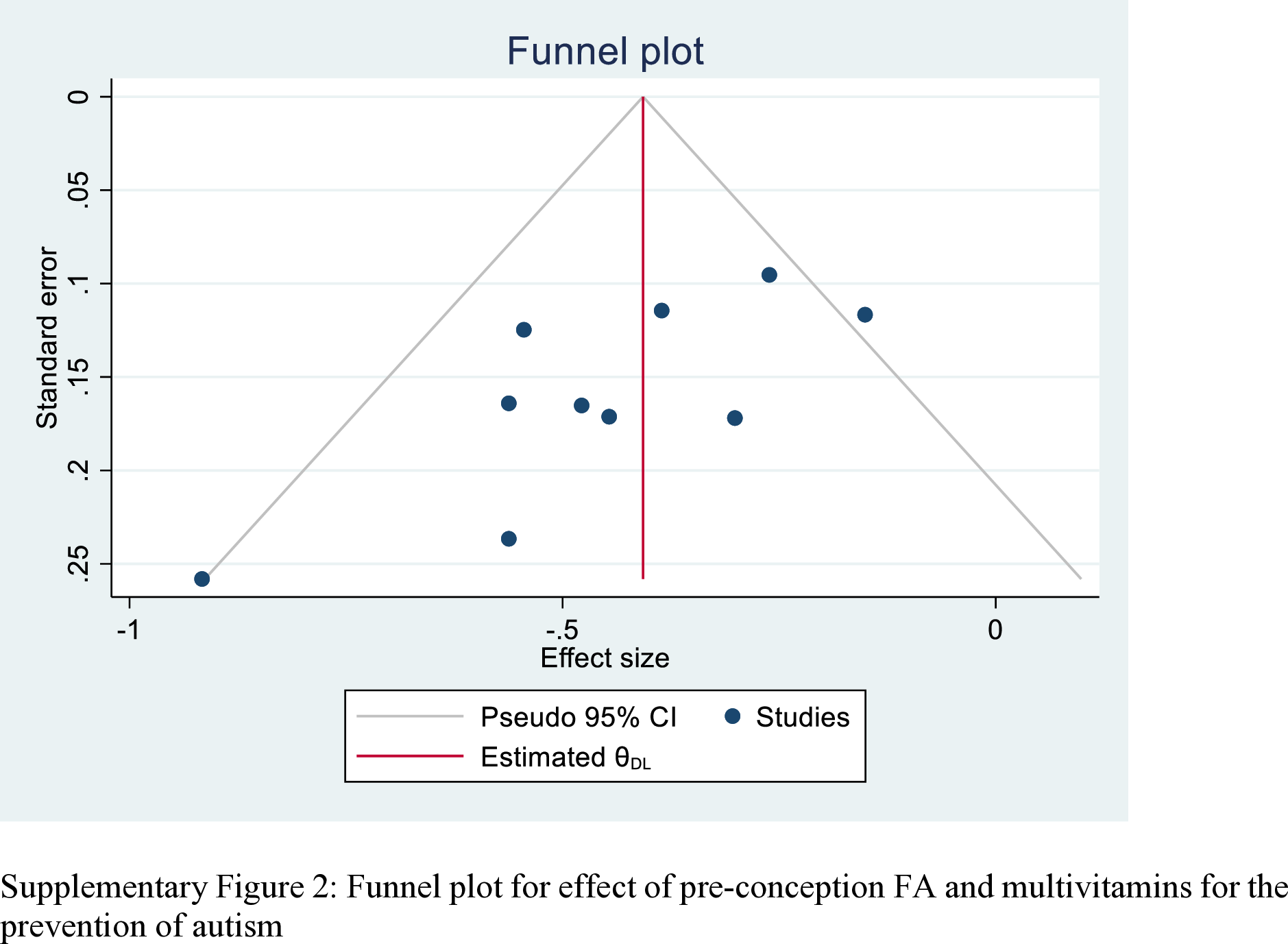

Supplement: S2 Fig — (TIF) [file pone.0334852.s002.tif]
