# Supplementary material for: The association between maternal prenatal folic acid and multivitamin supplementation and autism spectrum disorders in offspring: An umbrella review
Source: PLoS One. 2025 Nov 18;20(11):e0334852. doi: 10.1371/journal.pone.0334852 (PMC12626298; doi:10.1371/journal.pone.0334852)
Supplement: S1 Table — (DOCX) [file pone.0334852.s003.docx]

**Supplementary Table 1:** Search strategy used for one of the databases

| Medline/PubMed | Search terms | MeSH (sub-terms in MeSH) |
| --- | --- | --- |
| #1 | Preconception  pre-conception  pre-natal | Preconception |
| #2 | Multivitamin  Vitamin  Mineral  Micronutrient  Antioxidant  Anti-oxidant  Diet | Multivitamin |
| #3 | Neural Tube Defect  NTD  Birth Defect  Congenital Defect  Congenital Anomaly  Anencephaly,  Spinal bifuda  Spinal oculta  Encephalocele  Meningomiocele  Meningocele  Neuro interic cyst  Acrania  Xencephale  Spinal dysraphism | Neural Tube Defect |
| #4 | Meta-analysis  Systematic review  Review | Review |
| #5 | Global  World wide |  |
| #1 AND #2 AND #3 AND #4 AND #5 | | |
