# Supplementary material for: The association between maternal prenatal folic acid and multivitamin supplementation and autism spectrum disorders in offspring: An umbrella review
Source: PLoS One. 2025 Nov 18;20(11):e0334852. doi: 10.1371/journal.pone.0334852 (PMC12626298; doi:10.1371/journal.pone.0334852)
Supplement: S2 Table — (DOCX) [file pone.0334852.s004.docx]

**Supplementary Table 2**: Shows quality appraisal of included SRM using AMSTAR-2 checklist

| First Author | Questions and inclusion criteria (PICO) | Protocol | Study design | Comprehensive search | Study selection | Data Extraction | Excluded studies justification | Included studies details | Risk of Bias (ROS) | Funding Source | Statistical Methods | ROB on meta-analysis | ROB in individual studies | Explanation on heterogeneities | Publication bias | Conflict of interest | Overall quality |
| --- | --- | --- | --- | --- | --- | --- | --- | --- | --- | --- | --- | --- | --- | --- | --- | --- | --- |
| Guo B-Q, et al. (10) | Yes | No | Yes | Yes | Yes | Yes | Yes | Yes | Yes | Yes | Yes | No | No | Yes | No | Yes | High |
| Guo B-Q, et al. (15) | Yes | No | Yes | Yes | Yes | Yes | Yes | Yes | Yes | Yes | Yes | No | Yes | Yes | Yes | Yes | High |
| Li M, et al. (11) | Yes | No | Yes | Yes | Yes | Yes | Yes | Yes | Yes | Yes | Yes | No | Yes | No | No | Yes | Low |
| Friel C, et al. (16) | Yes | Yes | Yes | Yes | Yes | Yes | Yes | Yes | Yes | Yes | Yes | Yes | Yes | No | Yes | Yes | High |
| Liu X, et al. (12) | Yes | No | Yes | Yes | Yes | Yes | Yes | Yes | Yes | Yes | Yes | Yes | Yes | Yes | Yes | Yes | High |
| Wang M, et al. (27) | Yes | No | Yes | Yes | Yes | Yes | Yes | Yes | Yes | Yes | Yes | No | Yes | Yes | Yes | Yes | High |
| Iglesias VL et al (13) | Yes | No | Yes | Yes | Yes | Yes | Yes | Yes | Yes | Yes | Yes | No | Yes | Yes | Yes | Yes | High |
| Chen H (14) | Yes | Yes | Yes | Yes | Yes | Yes | No | Yes | No | Yes | Yes | No | Yes | Yes | Yes | Yes | Low |
