# Supplementary material for: The association between maternal prenatal folic acid and multivitamin supplementation and autism spectrum disorders in offspring: An umbrella review
Source: PLoS One. 2025 Nov 18;20(11):e0334852. doi: 10.1371/journal.pone.0334852 (PMC12626298; doi:10.1371/journal.pone.0334852)
Supplement: S3 Table — (DOCX) [file pone.0334852.s005.docx]

**Supplementary Table 3:** Quality Assessment Using the GRADE Framework of Each Pooled Analysis

| **Outcome assessed** | **Number of included meta-analyses** | **Number of individual studies** | **Number of cases/total sample size** | **Risk of bias assessment** | **Inconsistency/Heterogeneity** | **Indirectness** | **Imprecision** | **Publication bias** | **Summary of findings** | **GRADE level of /certainty of evidence** |
| --- | --- | --- | --- | --- | --- | --- | --- | --- | --- | --- |
| Folic acid supplementation for autism prevention | 5 | 50 | >13310/2727787 (No downgrade, no serious limitations) | More than 3/4th of the questions ‘‘yes’’ and 1/4th is ‘‘unclear’’ or ‘‘no’’ on AMSTAR | I^2^=49.89% (Moderate heterogeneity)- (Downgrade 1 level)  P value=0.11 | No serious indirectness | No serious imprecision | No publication bias was detected (Egger test P=0.0935 | (OR=0.70, 95% CI=0.60–0.81) | Highly suggestive |
| Multivitamin supplementation for autism prevention | 3 | 35 | >21436/2174123 (No downgrade, no serious limitations) | More than 3/4th of the questions is ‘‘yes’’ and 1/4th are ‘‘unclear’’ or ‘‘no’’ on AMSTAR | I^2^=2.34% (No heterogeneity)- (Downgrade 1 level)  P value=0.39 | No serious indirectness | No serious imprecision | No publication bias was detected (Egger test P=0.0935) | (OR=0.61, 95% CI=0.52–0.73) | Highly suggestive |

**Abbreviations**: ROB, risk of bias; AMSTAR, the AMSTAR quality assessment tool (49-51).
