# Supplementary material for: The association between maternal prenatal folic acid and multivitamin supplementation and autism spectrum disorders in offspring: An umbrella review
Source: PLoS One. 2025 Nov 18;20(11):e0334852. doi: 10.1371/journal.pone.0334852 (PMC12626298; doi:10.1371/journal.pone.0334852)
Supplement: Attachment — Submitted filename: plosone review.docx [file pone.0334852.s008.docx]

**Pre-conception folic acid and multivitamin supplementation for prevention of autism in offspring: An umbrella review of Systematic Review and Meta-analysis**

Thank you for the opportunity to review this umbrella review. It addresses an important question, however it is not effective in the message it endeavours to deliver. There are some major grammatical and formatting errors throughout, specifically the results tables. I am not convinced that this manuscript adds to what is already known regarding supplementation and outcomes of ASD. I believe there could be more substantial analysis, more in depth, and a greater discussion regarding the results and the implications.

**Abstract**

Result : please explain SRM.

‘*Subgroup analysis by type of supplement revealed that the pooled effect of either folic acid or multivitamin supplementation for the prevention of autism was found to be 0.69(0.58, 0.80) in the multivitamin group, while this estimate is 0.70(0.55,0.85) among studies with folic acid supplementation*.’ Please give units/meaning to this figures, and rephrase this to avoid such repetition, for example Subgroup analysis by type of supplement revealed that the pooled effect of either folic acid alone or multivitamin supplementation for the prevention of autism was found to be 0.70(0.55,0.85) and 0.69(0.58, 0.80) respectively.

*‘with a slightly higher (1%) percentage of reduction in autism’* please rephrase to: with a 1% greater reduction in autism’.

**Introduction**

*‘Ongoing studies are most frequently considered in environmental risk* factors’ can you reference this please?

‘*Preconception and prenatal maternal nutrition are among the modifiable risk factors of ASD’* please add a reference.

‘*The current review explored that during pregnancy, maternal exposure to Multivitamins and folic acid supplementation significantly reduces the likelihood of ASD in offspring.’* Please rephrase – the current review explores maternal exposure to multivitamins and folic acid supplementation during pregnancy and its association with ASD in offspring’ or something similar.

‘*Folic is water soluble’* please add folic **acid.**

*‘Various folate forms participate in essential reactions, like DNA methylation and replication’* – needs a reference.

‘FA is metabolized differently to naturally occurring forms of folates with a bioavailability of approximately 70% higher. 7’ Please also rephrase this sentence as it is not clear what the meaning of it is.

Folic acid is not abbreviated to FA prior to this – please use folic acid (FA) at first use, and then continue to use FA throughout. Please be consistent in either using the full term of folic acid or the abbreviation FA.

‘*Since folate is essential for metabolism and nervous system function and as a precursor of S-adenosyl-methionine (SAM), the universal methyl donor that is essential for cellular methylation of lipids, proteins, nucleic acid, and metabolites, inadequate availability affects the gene expression in the neurodevelopmental process which may have an association between maternal folate level during pregnancy and ASD mediated by DNA methylation. 8, 9’* please shorten this sentence or break it into multiple sentences. It is quite long as it is and difficult to follow for the reader.

**Methods**

SRM is introduced without full explanation – please write the term in full before using it as an abbreviation.

*‘From checking PROSPERO, this umbrella review needed to be registered.’* Please remove.

*‘We searched systematic review and meta-*analysis’ please rephrase to We searched **for** systematic review**s** and meta-analys**es**

**‘***in global context on 23/10/ 2023 G. C. Were searched SRM which reported the effect of preconception folic acid and/or multivitamin supplementation for prevention of autism in global context using PICO frameworks’* please rephrase as this is incorrect grammar and difficult to decipher what you are saying, specifically at the mention of G.C. and we ‘searched SRM which reported…’

PICO only needs to be mentioned once.

*‘was also performed’* remove also.

*‘The quality scoring was done out of 11, with scores 8–11, 4–7, and < 3 indicating high, medium, and low qualities’* Please re-organize this sentence to start from low, medium and high so that it reads chronologically.

There is only mention of investigators screening the title and abstracts, what about the full text screening? How were disagreements resolved?

*‘After the data was extracted using Microsoft Excel format, we imported the data to STATA version 17.0 statistical software for further analysis’* please reduce this to ‘STATA v17.0 was used for statistical analyses.

**Results**

*‘After duplication was removed’* please correct the grammar so that it reads ‘ after duplicates were removed’.

The flow chart needs clarification – Were the 721 duplicates? Or that’s how many remained after duplicate removal? It needs to be cleaned in terms of layout also – the boxes are not in line, the arrows are not in line, the text in the blue box doesn’t fit. It needs to be cleaned.

Please highlight further the ‘quality reasons’ for exclusion in your methods.

‘*Included SRM included from five to 16 studies’* please rephrase to avoid repetition of included, it is confusing.

‘….0.57, 95% CI: 0.41, 0.78) 15 to 0.91, 95% CI: 0.73, 1.13)’ please fix the use of brackets.

*‘The random-effects model analysis from those studies revealed that, the pooled effect of either folic acid or multivitamin supplementation’* remove the comma or rephrase please.

*‘This indicates prenatal folic acid and /or folic acid supplementation was associated’* please correct this – there is no distinction between the and/or folic acid mentioned.

*‘Subgroup analysis was done through stratified by the type of supplementation. Based on this..’* please rephrase. Remove ‘Based on this’.

*‘Based on this, the pooled effect of either folic acid or multivitamin supplementation for the prevention of autism was found to be 0.69(0.58, 0.80) in in multivitamin group, while this estimate is 0.70(0.55,0.85) among studies with folic acid supplementation’* remove the second ‘in’ and rephrase so that each supplementation type is only mentioned once – there is too much repetition. Please give meaning to the figures – there is no unit or indication as to what these figures represent.

*‘which indicated that, the absence of publication bias’* rephrase to ‘which indicates the absence of publication bias’.

It is evident from your results that you are using screenshots from the STATA output of results. Please enter these into appropriately formatted tables, only highlighting the necessary information as opposed to the excess of information and the many decimal points from the screenshot. It is inappropriate.

‘*Our pooled estimated effect of preconception folic acid and multivitamin for the prevention of autism varied from 0.68 (0.57– 0.78) to 0.71 (0.62–0.81) after the deletion of a single study (Fig 5).’* Use the full-term figure as opposed to fig, and give meaning to the numbers.

I think it would be interesting to ascertain if the multivitamins used in the studies contained folic acid. The introduction highlighted the lack of consensus regarding dose, frequency and timing of supplementation, but this was not addressed in the results.

There is little mentioned regarding the quality of the reviews included in the results. Please rectify.

As this is a global context, I think it would be interesting to see exactly what countries were included. I would imagine a difference would occur depending on socioeconomic status of the countries included, among other environmental factors.

**Discussion**

*‘increases the risk of (NTDs)’* please include Neural Tube Defects.

*‘insufficient folic acid intake during the early stages of pregnancy can result in (NTDs),’* remove brackets around NTDs.

Your discussion is brief, and focuses only on folic acid, when the review includes multivitamins. Please consider the numerous aspects at play here, the discussion to me does not consider all of the evidence and is very one-sided. Please broaden.

Is 7 studies considered substantial? Particularly when you started with >1000. I would have noted the low number of studies included.

You mention AMSTAR is used but there is no evidence of this use in your results.

*‘Despite these strengths the study also has few limitations: as the included studies were not from all countries and this may affect the generalizability of the pooled result’* this should be rephrased to improve grammar.

*‘despite the authors tried to reduce it through’* please rephrase to ‘despite the authors efforts to reduce it…’

*‘Participants who took multivitamin supplementation were associated with a slightly higher (1%) percentage of reduction in autism compared with those who took folic acid.’* Rephrase as highlighted previously.

I am not convinced that this umbrella review really adds to what is already known. Please elaborate in your discussion, highlighting the importance of this work and how it addresses the gaps in our existing knowledge. I feel as though the introduction leads the reader to believe that this will look at importance dose quantity, frequency, supplementation timing, etc., which is not the case.
