# Supplementary material for: The association between maternal prenatal folic acid and multivitamin supplementation and autism spectrum disorders in offspring: An umbrella review
Source: PLoS One. 2025 Nov 18;20(11):e0334852. doi: 10.1371/journal.pone.0334852 (PMC12626298; doi:10.1371/journal.pone.0334852)
Supplement: Attachment — Submitted filename: Response to reviwers comments_BD.docx [file pone.0334852.s010.docx]

**Response to reviewers' and editors’ comments**

Dear Editors and Reviewers,

We would like to express our sincere gratitude for the time and effort you have dedicated to reviewing our manuscript and providing thoughtful feedback. Your insightful suggestions have been instrumental in enhancing the quality of our work. We have carefully considered and incorporated your recommendations into the revised manuscript, with all changes highlighted in yellow for your convenience.

Below, we have addressed each of the reviewers' comments point by point:

**Reviewer #1**

**Reviewer comment:** Thank you for the opportunity to review this umbrella review. It addresses an important question; however, it is not effective in the message it endeavours to deliver. There are some major grammatical and formatting errors throughout, specifically the results tables. I am not convinced that this manuscript adds to what is already known regarding supplementation and outcomes of ASD. I believe there could be more substantial analysis, more in-depth, and a greater discussion regarding the results and the implications.

**Authors' response:** We greatly appreciate your constructive feedback. In response, we have corrected the grammatical and formatting issues, particularly in the results tables, and made revisions throughout the manuscript to improve clarity and consistency. Additionally, we have expanded the discussion section to offer a more comprehensive analysis of the results and their implications. All changes in the manuscript are highlighted in yellow, and we have addressed each point in detail in this response letter.

**Abstract**

**Reviewer comment:** Result: please explain SRM.

**Authors' response:** Thank you for pointing this out. We have revised the manuscript to clarify SRM in the methods section **(see Abstract: Methods, Page 2, line number 35)**

*“Systematic reviews and meta-analyses (SRMs)”*

**Reviewer comment:** ‘Subgroup analysis by type of supplement revealed that the pooled effect of either folic acid or multivitamin supplementation for the prevention of autism was found to be 0.69(0.58, 0.80) in the multivitamin group, while this estimate is 0.70 (0.55,0.85) among studies with folic acid supplementation.’ Please give units/meaning to this figure, and rephrase this to avoid such repetition, for example Subgroup analysis by type of supplement revealed that the pooled effect of either folic acid alone or multivitamin supplementation for the prevention of autism was found to be 0.70(0.55,0.85) and 0.69(0.58, 0.80) respectively.

**Authors' response:** Thank you for your feedback. We have added units to the figure, revised this section accordingly, and rephrased the sentence for clarity and to avoid repetition **(see Abstract: Results, Page 2, lines 45-48).**

*“Subgroup analysis by supplement type showed that maternal prenatal multivitamin supplementation reduced the risk of ASD by 34% (RR = 0.66, 95% CI: 0.55–0.80; GRADE: highly suggestive), while folic acid supplementation was associated with a 30% reduction in ASD risk (RR = 0.70, 95% CI: 0.60–0.83; GRADE: highly suggestive)”*

**Reviewer comment:** ‘with a slightly higher (1%) percentage of reduction in autism’ please rephrase to: with a 1% greater reduction in autism’.

**Authors' response:** Thank you for this suggestion. We have deleted this sentence from the Abstract section.

**Introduction**

**Reviewer comment:** ‘Ongoing studies are most frequently considered in environmental risk factors’ can you reference this please?

**Authors' response:** We have added the appropriate reference to support the statement. **(see Introduction: Page 3, line number 60-63).**

*“The etiologic and risk factors have been proposed to be a single or a group of genetic mutations and environmental influences (4). Ongoing studies are most frequently considered in environmental risk factors (5), and prenatal maternal nutrition are among the modifiable risk factors for ASD (6).”*

**Reviewer comment:** ‘Preconception and prenatal maternal nutrition are among the modifiable risk factors of ASD’ please add a reference.

**Authors' response:** We have added the reference to support this claim. **(see Introduction: Page 3, line number 61-63).**

*“prenatal maternal nutrition are among the modifiable risk factors for ASD (6)”.*

**Reviewer comment:** ‘The current review explored that during pregnancy, maternal exposure to Multivitamins and folic acid supplementation significantly reduces the likelihood of ASD in offspring.’ Please rephrase – the current review explores maternal exposure to multivitamins and folic acid supplementation during pregnancy and its association with ASD in offspring’ or something similar.

**Authors' response:** We have revised the sentence as suggested **(See Page 13, Line 282-283).**

*“The current review explored maternal exposure to multivitamins and folic acid supplementation during pregnancy and its association with ASD in offspring.”*

**Reviewer comment:** ‘Folic is water soluble’ please add folic acid.

**Authors' response:** Thank you for this correction. We have used folic acid consistently throughout the manuscript.

**Reviewer comment:** ‘Various folate forms participate in essential reactions, like DNA methylation and replication’ – needs a reference.

**Authors' response:** We have rephrased this sentence and added relevant references to support this sentence **(see Introduction: Page 3, line number 64-67).**

*“Folic acid supports DNA methylation and epigenetic regulation, both critical for neurodevelopment, and aids in neural tube formation, protecting against defects associated with neurodevelopmental disorders (7, 8)”.*

**Reviewer comment:** ‘FA is metabolized differently to naturally occurring forms of folates with a bioavailability of approximately 70% higher. 7’ Please also rephrase this sentence as it is not clear what the meaning of it is.

**Authors' response:** Thank you for pointing this out. We have removed the confusing sentence.

**Reviewer comment:** Folic acid is not abbreviated to FA prior to this – please use folic acid (FA) at first use and then continue to use FA throughout. Please be consistent in either using the full term of folic acid or the abbreviation FA.

**Authors' response:** Thank you for pointing this out. We have now used the full term “made “folic acid” throughout the manuscript.

**Reviewer comment:** ‘Since folate is essential for metabolism and nervous system function and as a precursor of S-adenosyl-methionine (SAM), the universal methyl donor that is essential for cellular methylation of lipids, proteins, nucleic acid, and metabolites, inadequate availability affects the gene expression in the neurodevelopmental process which may have an association between maternal folate level during pregnancy and ASD mediated by DNA methylation. 8, 9’ please shorten this sentence or break it into multiple sentences. It is quite long as it is and difficult to follow for the reader.

**Authors' response:** We have revised this sentence by breaking it into two shorter, clearer sentences for better readability **(see Introduction: Page 3, line number 64-67).**

*“Folic acid supports DNA methylation and epigenetic regulation, both critical for neurodevelopment, and aids in neural tube formation, protecting against defects associated with neurodevelopmental disorders (7, 8).”*

**Methods**

**Reviewer comment:** SRM is introduced without full explanation – please write the term in full before using it as an abbreviation.

**Authors' response:** Thank you, this has been corrected. **(see Abstract: Methods, Page 2, line number 35)**

**Reviewer comment:** ‘From checking PROSPERO, this umbrella review needed to be registered.’ Please remove.

**Authors' response:** Thank you. We have removed this statement as requested.

**Reviewer comment:** ‘We searched systematic review and meta-analysis’ please rephrase to We searched for systematic review**s** and meta-analyses.

**Authors' response:** Thank you for this suggestion. We have rephrased this sentence for clarity. **(see Abstract: Methods, Page 2, lines 35-36)**

***“****Articles were searched in PubMed, Scopus, Web of Science, and Google Scholar”*

**Reviewer comment:** **‘**in global context on 23/10/ 2023 G. C. Were searched SRM which reported the effect of preconception folic acid and/or multivitamin supplementation for prevention of autism in global context using PICO frameworks’ please rephrase as this is incorrect grammar and difficult to decipher what you are saying, specifically at the mention of G.C. and we ‘searched SRM which reported…’

**Authors' response:** We have revised this sentence for clarity and fixed the grammatical issues.

**(see Methods, Page 4, line number 91-94)**

*“We performed a comprehensive systematic search for SRMs that assessed the association between prenatal folic acid and/or multivitamin supplementation and the risk of ASD on a global scale, using the PICO (Population, Intervention, Comparison, and Outcome) framework.”*

**Reviewer comment:** PICO only needs to be mentioned once.

**Authors' response:** We have corrected this and removed the redundant mention of PICO.

**Reviewer comment:** ‘was also performed’ remove also.

**Authors' response:** Thanks, and we have removed it.

**Reviewer comment:** ‘The quality scoring was done out of 11, with scores 8–11, 4–7, and < 3 indicating high, medium, and low qualities’ Please re-organize this sentence to start from low, medium and high so that it reads chronologically.

**Authors' response:** We have revised the sentence as requested **(see Methods, Page 6, line number 134-135).**

*“The quality was scored out of 11, with scores < 3, 4–7, and 8–11 indicating low, moderate, and high qualities, respectively.”*

**Reviewer comment:** There is only mention of investigators screening the title and abstracts, what about the full-text screening? How were disagreements resolved?

**Authors' response:** Thank you for this valuable comment. We have clarified that the same procedures were followed for both title/abstract screening and full-text screening, and we have described how disagreements were resolved during this process **(see Methods, Page 5, line number 109-112).**

*“Disagreements in both title/abstract and full-text screening were addressed through structured discussions during consensus meetings, where team members collaboratively reviewed the criteria and reached a resolution based on predefined guidelines.”*

**Reviewer comment:** ‘After the data was extracted using Microsoft Excel format, we imported the data to STATA version 17.0 statistical software for further analysis’ please reduce this to ‘STATA v17.0 was used for statistical analyses.

**Authors' response:** We have revised the sentence to make it more concise, as suggested.

**(see Methods, Page 7, line number 150).**

*“STATA v17.0 was used for statistical analyses”*

**Results**

**Reviewer comment:** ‘After duplication was removed’ please correct the grammar so that it reads ‘ after duplicates were removed’.

**Authors' response:** We have corrected this as requested. **(see Results, Page 8, line number 177-178).**

*“After duplicates were removed, 667 articles remained to be screened for title and abstract.”*

**Reviewer comment:** The flow chart needs clarification – Were the 721 duplicates? Or that’s how many remained after duplicate removal? It needs to be cleaned in terms of layout also – the boxes are not in line, the arrows are not in line, the text in the blue box doesn’t fit. It needs to be cleaned.

**Authors' response:** We have clarified this to avoid confusion. 721 were duplicates, and 667 studies remained for title and abstract screening. We have also revised the flowchart for better clarity and layout, ensuring that the boxes and arrows are aligned and that the text fits appropriately. **(see Results, Page 8, line number 176-180 and Figure 1).**

*“A total of 1388 reviews were identified; 1380 were from different databases, and eight were from other sources.* *After duplicates were removed, 667 articles remained to be screened for title and abstract. After title and abstract screening, 142 studies remained for full-text review. Finally, after excluding 134 (using eligibility criteria), 8 SRMs, comprising 101 primary studies with 3,029,208 participants were included in the final analysis (Figure 1).”*

**Reviewer comment:** Please highlight further the ‘quality reasons’ for exclusion in your methods.

**Authors' response:** We have provided more details on the quality reasons for exclusion. Studies were excluded due to inadequate methodology, such as failure to clearly define exposure and treatment groups, and lack of regression or analytical analysis, which hindered meaningful estimates **(see Results, Page 8, line number 191-194).**

*“The most common reasons for a low-quality rating based on the AMSTAR-2 checklist are: failure to register the protocol in PROSPERO (10-12, 14, 15, 29), absence of an evaluation of the risk of bias in the meta-analysis (11-15, 29), insufficient explanation of heterogeneity (15, 30), and failure to assess publication bias (14, 15) (Table S1).”*

**Reviewer comment:** ‘Included SRM included from five to 16 studies’ please rephrase to avoid repetition of included, it is confusing.

**Authors' response:** Thank you We have revised the sentence **(see Results, Page 8, line number 185).**

*“The included SRMs ranges from five (14) to 20 (15) primary studies.”*

**Reviewer comment:** ‘….0.57, 95% CI: 0.41, 0.78) 15 to 0.91, 95% CI: 0.73, 1.13)’ please fix the use of brackets.

**Authors' response:** Thank you for the feedback, this has been corrected. **(see Results, Page 9, line number 202-203).**

*“The effect estimates from these included studies ranges from 0.57 (95% CI: 0.41, 0.78)(10) to 0.91 (95% CI: 0.73, 1.13) (14).”*

**Reviewer comment:** ‘The random-effects model analysis from those studies revealed that, the pooled effect of either folic acid or multivitamin supplementation’ remove the comma or rephrase please.

**Authors' response:** Thank you for the suggestion. We have removed the comma and rephrased the sentence for clarity. **(see Results, Page 9, line number 203-205).**

*“The random-effects model analysis revealed a 30% reduced risk of ASD (RR = 0.70, 95% CI: 0.62–0.78) in offspring of mothers who took folic acid and/or multivitamin supplementation during prenatal care, compared to those who did not.*

**Reviewer comment:** ‘This indicates prenatal folic acid and /or folic acid supplementation was associated’ Please correct this – there is no distinction between the and/or folic acid mentioned.

**Authors' response:** We appreciate your comment. The second mention of folic acid was intended to refer to multivitamin supplementation, and we have clarified this distinction in the revised text. **(see Results, Page 9, line number 203-205).**

*“The random-effects model analysis revealed a 30% reduced risk of ASD (RR = 0.70, 95% CI: 0.62–0.78) in offspring of mothers who took folic acid and/or multivitamin supplementation during prenatal care, compared to those who did not.*

**Reviewer comment:** ‘Subgroup analysis was done through stratified by the type of supplementation. Based on this..’ please rephrase. Remove ‘Based on this’.

**Authors' response:** Thank you. We have rephrased the sentence as suggested. **(see Results, Page 9, line number 206-209).**

*“Subgroup analysis by supplement type showed that maternal prenatal multivitamin supplementation reduced the risk of ASD by 34% (RR = 0.66, 95% CI: 0.55–0.80; GRADE: highly suggestive), while folic acid supplementation was associated with a 30% reduction in ASD risk (RR = 0.70, 95% CI: 0.60–0.83; GRADE: highly suggestive) (Figure 2).”*

**Reviewer comment:** ‘Based on this, the pooled effect of either folic acid or multivitamin supplementation for the prevention of autism was found to be 0.69(0.58, 0.80) in in multivitamin group, while this estimate is 0.70(0.55,0.85) among studies with folic acid supplementation’ remove the second ‘in’ and rephrase so that each supplementation type is only mentioned once – there is too much repetition.

**Authors' response:** We have revised the sentence to reduce repetition and correct the phrasing, as requested. We have rephrased the sentence as suggested. **(see Results, Page 9, line number 206-209).**

*“Subgroup analysis by supplement type showed that maternal prenatal multivitamin supplementation reduced the risk of ASD by 34% (RR = 0.66, 95% CI: 0.55–0.80; GRADE: highly suggestive), while folic acid supplementation was associated with a 30% reduction in ASD risk (RR = 0.70, 95% CI: 0.60–0.83; GRADE: highly suggestive) (Figure 2).”*

**Reviewer comment:** Please give meaning to the figures – there is no unit or indication as to what these figures represent.

**Authors' response:** Thank you for the valuable comment. We have updated the figures to include clear descriptions to enhance the understanding of the data. **(see Results, Page 20, Figure 2).**

**Reviewer comment:** ‘which indicated that, the absence of publication bias’ rephrase to ‘which indicates the absence of publication bias’.

**Authors' response:** Thanks for the suggestion. We have revised the sentence accordingly.

**(see Results, Page 9, Line 216-217)**

*“The symmetrical funnel plots and Egger’s test provided no evidence of substantial publication bias among the included studies (P-value = 0.14).”*

**Reviewer comment:** It is evident from your results that you are using screenshots from the STATA output of results. Please enter these into appropriately formatted tables, only highlighting the necessary information as opposed to the excess of information and the many decimal points from the screenshot. It is inappropriate.

**Authors' response:** Thank you for your feedback. We have removed the STATA screenshots and replaced them with appropriately formatted tables that highlight only the relevant information, ensuring a cleaner presentation. **(see Results, Page 23, Supplementary Figure 1)**

**Reviewer comment:** ‘Our pooled estimated effect of preconception folic acid and multivitamin for the prevention of autism varied from 0.68 (0.57– 0.78) to 0.71 (0.62–0.81) after the deletion of a single study (Fig 5).’ Use the full-term figure as opposed to fig and give meaning to the numbers.

**Authors' response:** Thank you. We have used the full term "Figure" and provided clear explanations for the numbers, as requested.

**(see Results, Page 9, Line 212-214)**

*“The pooled sensitivity estimate for the association between maternal prenatal folic acid and multivitamin supplementation and offspring ASD ranged from 0.67 (95% CI: 0.61–0.74) (29) to 0.72 (95% CI: 0.64–0.81) (12) (Figure S1).”*

**Reviewer comment:** I think it would be interesting to ascertain if the multivitamins used in the studies contained folic acid. The introduction highlighted the lack of consensus regarding dose, frequency and timing of supplementation, but this was not addressed in the results.

**Authors' response:** We appreciate your suggestion. We have now included information about the folic acid content in the multivitamins used in the studies and discussed its potential impact on the results. This addition provides further context regarding the factors influencing the findings.

**Reviewer comment:** There is little mentioned regarding the quality of the reviews included in the results. Please rectify.

**Authors' response:** Thank you for your comment. We have now addressed the quality of the systematic reviews based on the AMSTAR-2 criteria. The majority were rated as high quality, with some rated as low quality. We have outlined the reasons for the low-quality ratings and included the detailed assessment in Table S1 **(see Results, Page 8, Line 189-194)**

*“**Based on AMSTAR-2 criteria, the majority of the SRMs (6 out of 8)(10-12, 14, 29, 30) were assessed as high quality, while 2 out of 8* *were rated as low quality (13, 15) (Table S1).* *The most common reasons for a low-quality rating based on the AMSTAR-2 checklist are: failure to register the protocol in PROSPERO (10-12, 14, 15, 29), absence of an evaluation of the risk of bias in the meta-analysis (11-15, 29), insufficient explanation of heterogeneity (15, 30), and failure to assess publication bias (14, 15) (Table S1).”*

**Reviewer comment:** As this is a global context, I think it would be interesting to see exactly what countries were included. I would imagine a difference would occur depending on socioeconomic status of the countries included, among other environmental factors.

**Authors' response:** Thank you for your insightful comment. While we have incorporated eight systematic reviews and meta-analyses, these reviews collectively encompass over 95 individual studies conducted in various countries worldwide. Additionally, we have acknowledged the potential influence of socioeconomic and environmental factors on the outcomes, offering a broader context for the global perspective of our findings.

**(See Strengths and limitations of the study: Page 12, Line 288-293)**

*“However, the study has some limitations. Firstly, the included studies were not representative of all countries, which may impact the generalizability of the pooled results. Furthermore, mild to moderate heterogeneity was observed, despite the authors' efforts to minimize it through a weighted inverse variance random-effects model and subgroup analyses.”*

**Discussion**

**Reviewer comment:** ‘increases the risk of (NTDs)’ please include Neural Tube Defects.

**Authors' response:** Thank you for your suggestion. We have updated the text to include "Neural Tube Defects" as per your request.

**See Discussion: Page 10, Line 242-243.**

*“Several existing studies have reported a link between reduced folate levels and an increased risk of neural tube defects (34-36).”*

**Reviewer comment:** ‘Insufficient folic acid intake during the early stages of pregnancy can result in (NTDs),’ remove brackets around NTDs.

**Authors' response:** Thank you. We have removed this sentence and revised the section.

**Reviewer comment:** Your discussion is brief, and focuses only on folic acid, when the review includes multivitamins. Please consider the numerous aspects at play here, the discussion to me does not consider all of the evidence and is very one-sided. Please broaden.

**Authors' response:** Thank you for your feedback. We have expanded the discussion to incorporate the role of multivitamins and considered the various factors influencing the results more comprehensively.

**Reviewer comment:** Is 7 studies considered substantial? Particularly when you started with >1000. I would have noted the low number of studies included.

**Authors' response:** Thank you for highlighting this point. We have removed this misleading sentence and revised the section.

**Reviewer comment:** You mention AMSTAR is used but there is no evidence of this use in your results.

**Authors' response:** Apologies for the oversight. We have now included the AMSTAR results in Table S1 as a supplementary file **(see Results, Page 8, Line 189-194)**

*“Based on AMSTAR-2 criteria, the majority of the SRMs (6 out of 8)(10-12, 14, 29, 30) were assessed as high quality, while 2 out of 8 were rated as low quality (13, 15) (Table S1). The most common reasons for a low-quality rating based on the AMSTAR-2 checklist are: failure to register the protocol in PROSPERO (10-12, 14, 15, 29), absence of an evaluation of the risk of bias in the meta-analysis (11-15, 29), insufficient explanation of heterogeneity (15, 30), and failure to assess publication bias (14, 15) (Table S1).”*

**Reviewer comment:** ‘Despite these strengths the study also has few limitations: as the included studies were not from all countries and this may affect the generalizability of the pooled result’ this should be rephrased to improve grammar.

**Authors' response:** Thank you for your suggestion. We have rephrased the sentence to improve grammar and clarity. **See strengths and limitations of the study: Page 12, Line 288-292.**

*“However, the study has some limitations. Firstly, the included studies were not representative of all countries, which may impact the generalizability of the pooled results. Furthermore, mild to moderate heterogeneity was observed, despite the authors' efforts to minimize it through a weighted inverse variance random-effects model and subgroup analyses.*

**Reviewer comment:** ‘despite the authors tried to reduce it through’ please rephrase to ‘despite the authors efforts to reduce it…’

**Authors' response:** Thank you for the feedback. We have made the suggested correction.

**See Strengths and limitations of the study: Page 12, Line 290-292.**

*“Furthermore, mild to moderate heterogeneity was observed, despite the authors' efforts to minimize it through a weighted inverse variance random-effects model and subgroup analyses”*

**Reviewer comment:** ‘Participants who took multivitamin supplementation were associated with a slightly higher (1%) percentage of reduction in autism compared with those who took folic acid.’ Rephrase as highlighted previously.

**Authors' response:** Thank you for highlighting this point. We have removed this sentence and revised the section.

**Reviewer #2:**

Dear Reviewer,

Thank you for your insightful suggestions and comments. We have carefully reviewed your feedback and incorporated it into the manuscript. We believe these revisions effectively address your concerns and enhance the quality of the manuscript. The changes have been clearly highlighted in yellow for easy reference. Please find our detailed responses to each of your queries below.

**Reviewer comment:** I do have some comments in the abstract section
¬ you should state introduction other than importance,

¬ DESIGN, SETTING, AND PARTICIPANTS these are already methods

**Authors' response:** Thanks for your feedback. We have revised these sections accordingly to better reflect the appropriate structure.

**(See Abstract: Background, Page 2, Line 29-33).**

- ***“Background:*** *Previous reviews have examined the association between maternal prenatal use of folic acid and multivitamin supplements and autism spectrum disorder (ASD) in children, but findings remain inconclusive.* *This umbrella review aims to synthesise the existing evidence on the association between prenatal folic acid and multivitamin supplementation and the risk of ASD in offspring.****”***
- We have deleted “DESIGN, SETTING, AND PARTICIPANTS” as indicated

**Reviewer comment:** Once you stated the number in result section so you should have to state general context of your finding in conclusion for example you got 69%( result) your conclusion might be (high or low based specific guideline to say high or low)

**Authors' response:** Thank you for your suggestion. We have revised the conclusion accordingly.

**(See Abstract: Conclusion, Page 2, Line 49-51).**

*“****Conclusion:*** *Maternal prenatal folic acid and multivitamin supplementation is associated with a reduced risk of ASD in offspring. These findings have important public health implications, suggesting that prenatal supplementation could help mitigate the risk of ASD in children.”*

**Reviewer comment:** Once in your result section you got prenatal folic acid and multivitamin supplementation for the prevention of autism globally is found to be 0.69, and in the conclusion prenatal folic acid and multivitamin supplementation was associated with a 31% reduction in autism. Am confusing with these what does it mean the finding 69% and 31% in conclusion, you see both sentences revealed reduction of autism.

**Authors' response:** Thank you for raising this point. We have revised the interpretation of the results to clarify the risk reductions. Specifically, a reduction of 0.67 corresponds to a 33% decrease in risk, a reduction of 0.60 corresponds to a 31% decrease, and a reduction of 0.61 corresponds to a 39% decrease. **(See Abstract: Result, Page 2, Line 42-48).**

***“****Results****:*** *Eight SRMs comprising 101 primary studies and over three million mother-offspring pairs were included. Prenatal folic acid or multivitamin supplementation was associated with a 30% reduced risk of ASD in offspring RR=0.70, 95% CI: 0.62, 0.78; GRADE: highly suggestive). Subgroup analysis by supplement type showed that maternal prenatal multivitamin supplementation reduced the risk of ASD by 34% (RR = 0.66, 95% CI: 0.55–0.80; GRADE: highly suggestive), while folic acid supplementation was associated with a 30% reduction in ASD risk (RR = 0.70, 95% CI: 0.60–0.83; GRADE: highly suggestive)”.*

**Reviewer comment:** DESIGN, SETTING, AND PARTICIPANTS: In this umbrella review using PRISMA guideline, PubMed, Embase, Scopus, Web of Sciences, Cochrane Database of Systematic Reviews, Scopus, and Google Scholar which reported the effects of folic acid and multivitamin supplementation for the prevention of autism in offspring were searched.
Does it prisma guideline a search engine?

**Authors' response:** Thanks for pointing this out. We have revised the text to accurately describe PRISMA as a guideline, not a search engine as follows in the method section of the abstract. **(See Abstract: Methods, Page 2, Line 34-36).**

*“This umbrella review followed the PRISMA guidelines to synthesise and report evidence from existing systematic reviews and meta-analyses (SRMs). Articles were searched in PubMed, Scopus, Web of Science, and Google Scholar. ”*

**Reviewer comment:** Key Points QUESTION: Does maternal folic acid and multivitamin supplement use before pregnancy decrease the risk of autism in offspring? FINDINGS In this umbrella review, seven systematic reviews and meta-analyses were included with 3,785,573 participants. A statistically significant association between maternal multivitamin and folic acid supplement use before pregnancy and reduced risk of autism in their offspring was observed. MEANING A reduced risk of autism spectrum disorder in children born to women who used multivitamins and folic acid supplements before pregnancy has important public health implications; possible mechanisms include epigenetic modifications.

**Authors' response:** We have removed this section and revised the abstract following the journal's formatting guidelines **(See Abstract: Page 2, Line 29-51).**

**Reviewer comment:** Is that seven systematic review with 3,785,573 participant your key findings? It is better to report the main findings that you got from 7 systematic review articles

**Authors' response:** Thank you for this helpful suggestion. We have revised this section to focus on the main findings derived from the seven systematic reviews and meta-analyses. **(See Abstract: Result, Page 2, Line 42-48).**

***“****Results****:*** *Eight SRMs comprising 101 primary studies and over three million mother-offspring pairs were included. Prenatal folic acid or multivitamin supplementation was associated with a 30% reduced risk of ASD in offspring RR=0.70, 95% CI: 0.62, 0.78; GRADE: highly suggestive). Subgroup analysis by supplement type showed that maternal prenatal multivitamin supplementation reduced the risk of ASD by 34% (RR = 0.66, 95% CI: 0.55–0.80; GRADE: highly suggestive), while folic acid supplementation was associated with a 30% reduction in ASD risk (RR = 0.70, 95% CI: 0.60–0.83; GRADE: highly suggestive)”.*

**Reviewer comment:** However, evidence derived from the previous reviews on the uses of folic acid and multivitamins for the prevention of ASD is inconclusive. Some studies concluded that there is no association between maternal folic acid supplementation during the prenatal period and the risk of ASD. 10, 11 On the other hand, other reviews revealed that there is a strong association between preconception and prenatal folic acid and multivitamin supplementations and the risk of autistic disorder. 12-15 Even for those who agree on the supplementations, there are inconsistencies in the timing (preconception or during pregnancy), the types of Nutrients (folic and Multivitamins), and each nutrient's dose. Therefore, this Umbrella review aims to explore the actual association between maternal supplementations with folic acid and multivitamins pre-conceptional and during pregnancy and the risk of ASD in offspring. Your interest is to do umbrella review, it is not elaborated more what is the important to do umbrella review, you only said “previous reviews on the uses of folic acid and multivitamins for the prevention of ASD is inconclusive.” What are the key features of umbrella review? What gaps were not answered by systematic review? You should have to clearly show the need to use umbrella review in the context of your title

**Authors' response:** Thank you for your valuable feedback. We have revised this section to better explain the rationale for conducting an umbrella review and to clarify the gaps left by previous systematic reviews. Umbrella reviews are particularly useful when synthesizing the findings of multiple systematic reviews and meta-analyses to provide an overarching perspective. In this case, despite over 101 primary studies and numerous systematic reviews and meta-analyses examining the relationship between maternal prenatal folic acid and multivitamin supplementation and ASD risk, the findings remain inconsistent. Some reviews suggest a protective effect, while others report no association. Furthermore, these reviews have not consistently addressed critical variables such as supplementation timing, and nutrient types. This umbrella review aims to resolve these inconsistencies by synthesizing the findings from existing reviews, thereby providing a clearer understanding of the role of folic acid and multivitamin supplementation in reducing ASD risk. **(See Introduction: Page 3&4, Line 72-82).**

*“**Early prevention is preferable to treatment, as no medication can address the core symptoms of ASD; available treatments focus on managing specific co-occurring conditions or symptoms. Maternal supplementation with folic acid and multivitamins before and during pregnancy is among the most accessible and cost-effective preventive measures. To date, seven systematic reviews and meta-analyses (SRMs) comprising 101 primary studies 101 have examined whether maternal prenatal folic acid supplementation reduces the risk of ASD in offspring. However, findings from previous reviews remain inconclusive.* *While some reviews have reported the association between prenatal folic acid and multivitamin supplementation and a lower risk of ASD (10-14), others have found no association (15, 16). This umbrella review aims to synthesise these inconsistent findings to clarify the potential role of prenatal folic acid and multivitamin supplementation in reducing ASD risk in offspring.”*

**Reviewer comment:** All published, unpublished, and systematic reviews and meta-analyses assess the effect of pre-conceptional folic acid and multivitamin supplementation for preventing autism in the offspring. We would have included studies reported as abstracts only and those with full-text manuscripts. We searched systematic review and meta-analysis from the following databases: PubMed, Embase, Scopus, Web of Sciences, Cochrane Database of Systematic Reviews, Scopus and Google Scholar in global context on 23/10/ 2023 G. C. Potentially eligible studies identified through database search (n=1380), Potentially eligible studies identified through other sources (n =8); How many articles have you identified through different search engine ( i.e how much was in Pub med, scops, unpublished article…etc )

**Author's response:** Thanks for your question. We have now provided a breakdown of how many articles were identified through each search engine/database, and we have updated this information in the revised manuscript **(See Results: Figure 1, Page 19).**

**Results and Discussion**

**Reviewer comment:** I did not see the implications of the study for policymakers and practitioners. Please provide unique, actionable strategies to address autism based on the findings.

**Authors' response:** Thanks for your valuable suggestion. We have now included specific implications for practice, policy, and future research based on the findings of the study **(See Implications for practice, policy, and future research: Page 13, Line 297-317).**

*“****Practice:*** *The findings emphasize the importance of prenatal folic acid and multivitamin supplementation as part of routine maternal care to reduce the risk of ASDs in offspring. Healthcare providers should encourage women to start supplementation during the pre-conception period and continue throughout pregnancy. This proactive approach can be integrated into preconception and prenatal care programs to promote maternal and fetal health.*

***Policy:*** *Policymakers should prioritize the inclusion of folic acid and multivitamin supplementation in national maternal health guidelines and public health initiatives. Public awareness campaigns are needed to educate women of reproductive age about the potential benefits of these supplements in reducing ASD risk. Subsidizing or ensuring the affordability of high-quality folic acid and multivitamin supplements, particularly in low-resource settings, can further enhance accessibility and adherence to these recommendations.*

***Research:*** *Future research should focus on conducting large-scale prospective cohort studies and randomized controlled trials (RCTs) to confirm and expand these findings. Key areas of investigation include exploring the molecular mechanisms behind the protective effects of folic acid and multivitamins against ASD, determining the optimal dosage, duration, and timing of maternal supplementation for maximum risk reduction, and assessing the roles of specific nutrients like vitamin D, zinc, and folate in mitigating ASD risk. Additionally, it is important to evaluate the impact of maternal supplementation across diverse populations and geographic regions to enhance generalizability and ensure culturally relevant interventions. Addressing these gaps will strengthen evidence to refine clinical guidelines and improve preventive strategies for ASD ”*

**Reviewer comment:** Ensure that the discussion goes beyond restating the results and connects them to broader public health and policy contexts.

**Authors' response:** Thanks for the feedback. We have expanded the discussion to link the results to broader public health and policy contexts, providing a more comprehensive interpretation of the findings. **(See Discussion: Page 11 &12, Line 232-281)**
